# Telephone and Web-Based Delivery of Healthy Eating and Active Living Interventions for Parents of Children Aged 2 to 6 Years: Mixed Methods Process Evaluation of the Time for Healthy Habits Translation Trial

**DOI:** 10.2196/35771

**Published:** 2022-05-26

**Authors:** Megan L Hammersley, Rebecca J Wyse, Rachel A Jones, Anthony D Okely, Luke Wolfenden, Simon Eckermann, Joe Xu, Amanda Green, Fiona Stacey, Sze Lin Yoong, Jacklyn Jackson, Christine Innes-Hughes, Vincy Li, Chris Rissel

**Affiliations:** 1 Early Start Faculty of the Arts, Social Sciences and Humanities University of Wollongong Wollongong Australia; 2 Illawarra Health and Medical Research Institute Wollongong Australia; 3 School of Health and Society Faculty of the Arts, Social Sciences and Humanities University of Wollongong Wollongong Australia; 4 School of Medicine and Public Health Faculty of Health and Medicine University of Newcastle Callaghan Australia; 5 Hunter New England Population Health Wallsend Australia; 6 Hunter Medical Research Institute New Lambton Heights Australia; 7 Priority Research Centre for Health Behaviour University of Newcastle Callaghan Australia; 8 School of Education Faculty of the Arts, Social Sciences and Humanities University of Wollongong Wollongong Australia; 9 Centre for Population Health New South Wales Ministry of Health St Leonards, Sydney Australia; 10 School of Health Sciences Swinburne University of Technology Hawthorn, Melbourne Australia; 11 Health Consult Sydney Australia; 12 College of Medicine and Public Health Flinders University Darwin Australia

**Keywords:** dietary intake, physical activity, screen time, sleep, movement behaviors, online, internet, telephone, mobile phone

## Abstract

**Background:**

Few translational trials have provided detailed reports of process evaluation results.

**Objective:**

This study reported on findings from a mixed methods process evaluation of a large translational trial comparing 2 remotely delivered healthy eating and active living interventions with an active control, targeting parents of young children.

**Methods:**

Mixed methods process evaluation data were collected as part of a 3-arm, partially randomized preference trial targeting parents of children aged 2 to 6 years from New South Wales, Australia. Recruitment strategies were assessed through the participant baseline questionnaire and a questionnaire completed by the health promotion staff involved in recruitment. Data on participants’ intervention preferences were collected at baseline and after the intervention. Intervention acceptability and demographic data were collected via a postintervention questionnaire (approximately 3 months after baseline), which was supplemented by qualitative participant interviews. Implementation data on intervention fidelity and withdrawal were also recorded. Differences in intervention acceptability, fidelity, and withdrawal rates between telephone and web-based interventions and between randomized and nonrandomized participants were analyzed. The significance level was set at *P*<.05 for all tests. The interview content was analyzed, key themes were drawn from participant responses, and findings were described narratively.

**Results:**

Data were collected from 458 participants in the baseline survey and 144 (31.4%) participants in the 3-month postintervention survey. A total of 30 participants completed the qualitative interviews. A total of 6 health promotion staff members participated in the survey on recruitment strategies. Most participants were recruited from Early Childhood Education and Care services. There was a broad reach of the study; however, better take-up rates were observed in regional and rural areas compared with metropolitan areas. Parents with a university education were overrepresented. Most participants preferred the web-based medium of delivery at baseline. There was high acceptability of the web-based and telephone interventions. Participants found the healthy eating content to be the most useful component of the modules (web-based) and calls (telephone). They regarded text (web-based) or verbal (telephone) information as the most useful component. A high proportion of participants completed the telephone intervention compared with the web-based intervention; however, more participants actively withdrew from the telephone intervention.

**Conclusions:**

This is one of the first studies to comprehensively report on process evaluation data from a translation trial, which demonstrated high acceptability of all interventions but a strong participant preference for the web-based intervention. This detailed process evaluation is critical to inform further implementation and be considered alongside the effectiveness outcomes.

## Introduction

The dietary habits and movement behaviors (physical activity, screen time, and sleep) of Australian children are well below the current recommendations and have deteriorated over time [1]. Parents are fundamental to establishing healthy behaviors during early childhood [2]. However, there are several barriers that can impede the involvement of parents in healthy lifestyle interventions for their children [3,4]. Remotely delivered interventions, such as telephone or web-based programs, have the potential to overcome these barriers, allow access regardless of location, and provide greater flexibility compared with face-to-face interventions. The Healthy Habits and Time2bHealthy remotely delivered parent-focused interventions have demonstrated efficacy in randomized controlled trials (RCTs). The *Healthy Habits* 4-week telephone-based intervention for parents of children aged 3 to 5 years showed a significant improvement in children’s fruit and vegetable intake [5]. The T*ime2bHealthy* 11-week web-based intervention demonstrated significant improvement in children’s discretionary food intake, parental nutrition self-efficacy, and child feeding practices [6]. These interventions were conducted under highly controlled conditions; however, more recently, both have been tested in a large translation trial (known as *Time for Healthy Habits*) to determine their effectiveness in a real-world context, with the potential for widespread implementation [7]. The *Time for Healthy Habits* study [8] investigated the effectiveness of the *Healthy Habits Plus* (enhanced *Healthy Habits*) telephone intervention and the *Time2bHealthy* web-based intervention against an active control group (receiving written materials). The protocol [8] and main outcomes of the *Time for Healthy Habits* translation study have been published elsewhere. Briefly, the study found that although there was no statistically significant difference between groups over time in relation to the primary outcome (children’s fruit and vegetable intake), there was a significant improvement over time among randomized participants receiving the telephone intervention for non–core food intake (secondary outcome) compared with participants receiving the control (written materials) [9]. There is a need to evaluate process data to further explore and explain these results so that any future decisions related to the potential scale-up of these interventions are fully informed. Process evaluations are critical to providing a comprehensive assessment of interventions alongside effectiveness testing, helping to determine how interventions work, whom they work for, how outcomes can be explained, and how interventions can be improved in the future, which are important considerations for policy and practice [10]. To date, a very limited number of studies have conducted process evaluations of children’s healthy eating and active living translation trials [11-13]. This study aimed to determine intervention acceptability, optimal recruitment strategies, participant intervention preference (ex ante and ex post), intervention fidelity, withdrawal rates, and participant representativeness concerning the target population.

## Methods

### Study Overview

This was a process evaluation of the *Time for Healthy Habits* study and comprised participant data from the main trial (collected at baseline and 3 months after the intervention) and data from 30 qualitative interviews across all intervention arms, conducted 1 to 10 months after the intervention. It also comprised data collected from the participant recruitment staff in the local health districts (LHDs) where the main trial was conducted. A detailed description of the protocol for the main effectiveness trial has been previously published [8]. Briefly, parents of children aged 2 to 6 years from New South Wales (NSW), Australia, were recruited. Parents were eligible if their children lived with them for at least 4 days per week on average and they spoke sufficient English to participate. The trial design was a 3-arm, partially randomized preference trial. Participants were initially provided with the option to choose their preferred delivery method (telephone, web-based, or written material) or to be randomized. This allowed us to establish the participants’ ex ante intervention preferences. The design was also thought to have higher initial participant acceptability than a traditional RCT, as participants may have been more willing to take part and complete the intervention if they knew that they were able to choose which intervention they received [14-19]. However, to ensure that sufficient participants were enrolled in the randomized arm of the study to establish intervention effectiveness via robust analysis, a stopping rule was applied to limit the number of participants who could choose their preferred intervention. After the application of the stopping rule, all participants were randomized in a 1:1:1 ratio. The 3 arms of the study included *Healthy Habits Plus* (a telephone intervention), *Time2bHealthy* (a web-based intervention), and an active control (written education materials). The specific features of the interventions are outlined in Table 1.

**Table 1:** Time for Healthy Habits intervention components.

Components	Time2bHealthy (web-based)	Healthy habits plus (telephone)	Active control (written materials)
Format	Web-based web application comprising 6 modules (1 per fortnight) over 3 months with email reminders	Six 20- to 30-minute fortnightly telephone calls over 3 months	2 factsheets fortnightly (10 in total) and 1 summary booklet over a period of 3 months
Content	Text, videos, practical activities, and quizzes Optional closed Facebook group	Verbal information Guidebook containing additional information and resources Pad of meal planner templates	Text information and images
Behavior change strategies	Barrier identification, goal setting, and self-monitoring	Barrier identification, goal setting, and self-monitoring	N/A^a^
Topics	Healthy eating, physical activity, screen time, and sleep	Healthy eating, physical activity, screen time, and sleep	Healthy eating, physical activity, screen time, and sleep

^a^N/A: not applicable.

### Ethics Approval

Ethics approval for the quantitative and qualitative aspects of this study was granted by the South Western Sydney LHD Human Research Ethics Committee (HE18/300), and site-specific approval was obtained from the human research ethics committees of the 5 LHDs involved in the study [8]. Acceptance was provided by the University of Newcastle Human Research Ethics Committee (H-2019-0188) and the University of Wollongong Human Research Ethics Committee (HE2019/207).

### Process Evaluation Data Collection and Measures

This mixed methods process evaluation reported data from the sources detailed in the following sections.

#### Preference and Demographic Characteristics

##### Baseline Questionnaire

Ex ante preferences were collected from all trial participants during the baseline interview (via telephone). Before the implementation of the stopping rule, participants were asked, “Do you have a strong preference for the way in which you receive healthy lifestyle advice or support about your child?” If they responded *yes*, they were then asked, “Would you prefer to receive healthy lifestyle advice or support via written information, telephone, or online” (with the order in which the interventions were stated to be randomized). After the implementation of the stopping rule, participants were still asked what their preferences would have been, although all participants were randomized from this point. Basic demographic data were also collected.

##### 3-Month Postbaseline Questionnaire

Ex post preference was ascertained from participants by the following question: “Having completed the program, would you have preferred for the information to be delivered in another way?” If they responded *yes*, they were asked, “In which format would you have preferred to receive the advice?” (response options included *online program*, *telephone counseling*, *educational materials*, *smartphone app*, *face-to-face*, *Skype*, *other*, and *do not know*).

#### Recruitment: Health Promotion Staff Surveys

The LHD staff (recruitment officers or other health promotion staff who were involved in the recruitment of parents to the study) completed a web-based questionnaire comprising 10 questions. The questions focused on recruitment strategies; recruitment challenges; and recommendations for future recruitment, including additional support. These questions are provided in Multimedia Appendix 1. In addition, the baseline participant questionnaire included a question on where they heard about the study.

#### Intervention Acceptability

##### 3-Month-Postbaseline Questionnaire

The postintervention (3 months after baseline) questionnaire included up to 27 process evaluation questions (depending on the intervention) and was completed over the telephone (for *Healthy Habits Plus* participants), on a web-based questionnaire (for *Time2bHealthy* and control group participants). A complete list of process evaluation questions can be found in Multimedia Appendix 2. The process evaluation questions were similar to those used previously in the process evaluation of the *Time2bHealthy* (web-based) RCT [6] and measured user acceptance of the content and modality of each intervention. Specifically, the participants were asked 5 questions about whether the intervention content was interesting, easy to understand, relevant to their family, worthwhile, and had information that they could act on. These questions used a Likert scale, with semantic anchors ranging from 1 (strongly disagree) to 5 (strongly agree). Each of these question responses was summed to attain an overall user acceptance score.

Participants were also asked about the appropriateness of the length and number of calls, web-based modules, or written resources. Furthermore, regarding the telephone and web-based interventions, they were asked to identify 1 intervention aspect that they found most useful (eg, for the telephone intervention: the guidebook, information provided verbally by the interviewer, goal setting, homework activities, and the meal planner templates; for the web-based intervention: information provided in text, videos, goal setting, and activities). Participants were also asked to identify the 1 call or module that they found most useful.

##### Participant Qualitative Interviews

In addition to the abovementioned questions asked in the 3-month postbaseline follow-up, a sample of participants from each of the 3 interventions was invited to participate in an additional telephone interview to further explore participants’ experiences. Participants were selected from a list of all those who participated in the interventions by March 2020, with the aim of interviewing 10 participants per intervention and the intention of capturing a targeted selection from metropolitan, rural, and regional areas (target of 16 metropolitan, 7 regional, and 7 rural participants); a combination of participants who had partially and fully completed the interventions; and a mix of participants from the randomized and preference arms of the study. Interviews were conducted by a research consulting company (Research Forum Consulting), which emailed participants, provided details about the interviews along with a participant information sheet, and informed them that they might receive a phone call to invite them to participate. When participants were phoned, they were provided with information about the interviews and asked to participate. Consent to proceed with the interview was obtained verbally, and participants provided consent for the interview to be audio recorded (optional). Questions were designed to capture participants’ overall and intervention-specific experiences (for telephone and web-based intervention participants only). Participants were asked about their initial expectations, intervention content, what they found most and least useful, length of the interventions, ease of completion, and engagement. A copy of the interview questions can be found in Multimedia Appendix 3. A total of 30 participants were interviewed (10 from each intervention group), which was anticipated to represent participants’ breadth of experience.

#### Fidelity and Withdrawal Rates: Intervention Implementation Data

Although the interventions were designed to be completed within 12 weeks, additional time was allocated (up to 20 weeks in total) to allow participants to complete the interventions by extending access to the web-based intervention and continuing to contact telephone participants to complete the intervention calls that had not yet been completed. Data were collected on the withdrawal of participants from the study, including whether the withdrawal was active (where the participant explicitly asked to be withdrawn) or passive (where the participant did not complete the intervention but did not ask to be withdrawn). We also determined the proportion of participants who completed each phase of the intervention; that is, the number of calls or modules completed.

### Data Analyses

Key themes were drawn from the responses to the health promotion staff survey questions, and findings were described narratively in relation to recruitment avenues used, recruitment barriers, and strategies that were most and least successful for recruitment. Participant responses to the 3-month postbaseline Likert scale questions on user acceptance were considered singularly and summed to produce a score from 5 to 25. Medians and IQR were determined. Kruskal-Wallis tests were used to assess differences in Likert scale responses to questions between all study groups and between randomized and nonrandomized participants. Participants’ qualitative interviews were audio recorded for all participants who provided consent (28/30, 93%) and then transcribed verbatim and deidentified. Detailed notes were obtained for those who did not consent to be recorded (2/30, 7%). The interview content was analyzed, and key themes were drawn from the participant responses. These findings were then described narratively in relation to the specific question domains, which were triangulated with the quantitative participant questionnaire data relating to the participant acceptability of the interventions. The number and percentage of preferences, randomized and total participants by study arm (telephone, web-based, and active control written materials) completing each module or call, and the number and percentage of active and total withdrawals (active and passive withdrawals) were calculated. Chi-square tests were used to assess differences in active and total withdrawal rates between interventions and differences between randomized and nonrandomized participants. Mann-Whiney *U* tests were used to assess differences in the number of calls or modules completed between the telephone and web-based study groups and differences between randomized and nonrandomized participants. Completion numbers and percentages (both completion at any time point and completion within 20 weeks) were calculated by the intervention group according to whether participants were randomized or nonrandomized. Chi-square tests were used to assess differences in completion of the intervention between the telephone and web-based intervention groups and differences between randomized and nonrandomized participants. In the first instance, these tests were based on the completion of modules, calls, and interventions within any time frame. They were then repeated based on the completion of the modules or calls within a 20-week time frame. The significance level was set at *P*<.05 for all tests. All analyses were conducted using SPSS Statistics for Windows (version 25.0; IBM Corp).

## Results

### Overview

**Figure 1:**
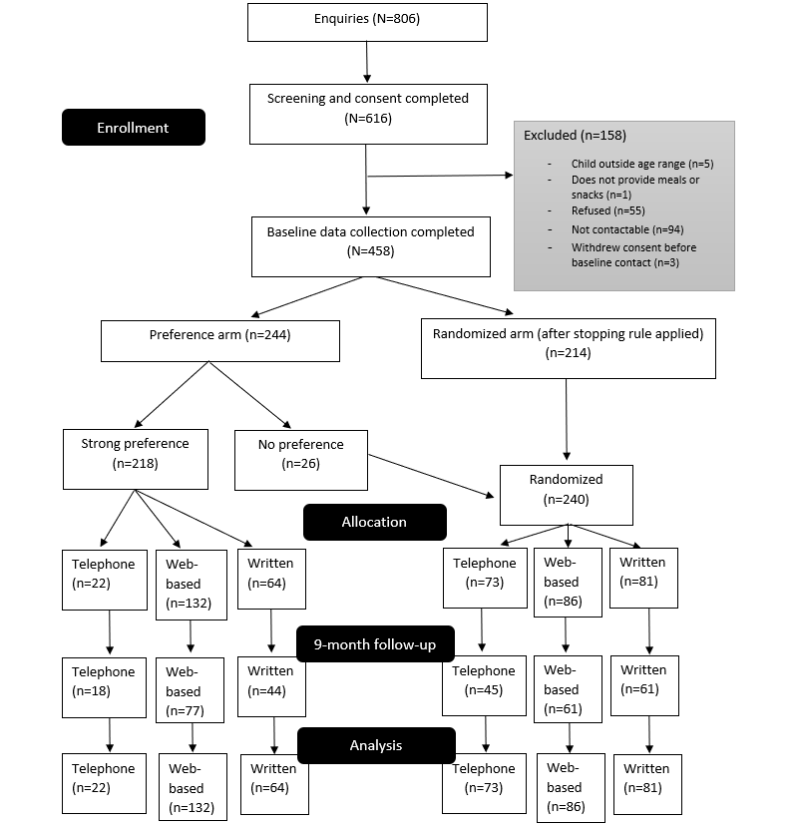
Time for healthy habits process evaluation CONSORT (Consolidated Standards of Reporting Trials) diagram.

Data collection was completed by 458 participants at baseline and 144 (31.4%) at postintervention (3 months after baseline), as shown in Figure 1. Of the 79 invited participants, 30 (38%) completed qualitative interviews, 10 (13%) from each intervention (including the active control group). Interviews for the qualitative study were conducted between March and April 2020, which was between 1 and 8 months after participants had completed one of the interventions (or the active control). Approximately 80% (24/30) were from metropolitan areas, and 20% (6/30) were from regional and rural areas (there was a higher proportion of metropolitan participants than the target). There was an even split of randomized to preference participants for the telephone (5:5) and active control (written materials; 4:6) participants; however, only one of the participants from the list of those who had completed the web-based intervention was randomized; thus, 30% (9/30) of participants interviewed from this intervention group were preference participants. Although attempts were made to include participants who had only partially completed the interventions, all participants who were interviewed had completed the interventions. All 5 LHDs targeted for recruitment were represented in the 6 responses to the health promotion staff survey.

### Recruitment

Participants were recruited for the main trial using established networks such as health promotion staff visits to Early Childhood Education and Care (ECEC) services; Child and Family Health nurses; and several other avenues, including playgroups and libraries, social media, media releases, the Playgroup Australia newsletter, the University of Wollongong Discovery Space (children’s museum) newsletter, and through health professionals. Most LHD-based recruitment works were conducted directly by recruitment staff employed within each LHD for the specific purpose of assisting with recruitment to this trial, with other recruitment activities (such as social media, newsletters, and bulk emails) conducted by a central project coordinator.

Participants reported finding out about the study from ECEC services or educators (188/458, 41%), Facebook or social media (87/458, 19%), and libraries (23/458, 5%). These findings were reiterated by the LHD recruitment staff, who indicated that ECEC services were the most successful channel, with some LHDs reporting that this was particularly effective when attending face to face, where there was an opportunity to talk to parents about the study directly. They also reported that they found library groups, playgroups, large events, and preschool sporting activities as efficient recruitment sites (likely because of being able to speak face to face with parents), and some (but not all) reported that mass mailouts to ECEC services were effective in recruiting parents.

The LHD health promotion staff reported via the survey that a facilitator of these successful recruitment channels was the ability to answer questions from parents face to face. They felt that parents were more likely to enroll in the study when there was someone present face to face, as many parents reported that they had seen a flyer about the study before enrolling:

Face-to-face conversation with parents, being able to explain the program to them in detail. Providing parents with the opportunity to ask questions and seek more info before signing up is important.

The LHD staff reported that the least effective recruitment channels were emails, posters, flyers, media releases, and large events that were not targeted to the age group. Although some staff members found mass mailouts to ECEC services effective, others did not. Some stated that mailouts were more successful when accompanied by a follow-up telephone call for the ECEC service:

Email alone—rarely received any form of contact/enquiry. Slightly better if emails were followed up with a phone call.

### Demographics

Considering the broad representativeness of the trial, there was a substantial representation of NSW-target LHDs in regional and rural areas; however, there was an underrepresentation of LHDs in metropolitan areas. The breakdown of participant proportions across the target LHDs is displayed in Table 2. Parent participants in the study were more likely to be female (441/458, 96.3%) compared with the general NSW population (50.7%). The mean age of participants (36.13, SD 4.92 years) was similar but perhaps slightly older than the general NSW parent population, given the median age of NSW first-time mothers and fathers (30.7 and 33.1 years, respectively) and that some parents already had older children. The mean age of child participants was 3.37 years (SD 1.16). A smaller percentage of participants spoke a language other than English at home (81/458, 17.7%) compared with the general NSW population (27%). The proportion of Aboriginal and Torres Strait Islander individuals (15/448, 3.3%) was similar to that of the NSW population (2.9%). There was a much higher proportion of university-qualified participants (322/458, 70.3%) than in the NSW population (23.4%). Over three-fourth of the participants had a household income higher than the NSW median household income [20].

**Table 2:** Number and proportion of participants recruited across target LHDs^a^ compared with drawing area (N=380).

LHD	Geographic area	Children in drawing area and proportion of total target drawing area^b^, n (%)	Participants recruited^c^, n (%)
Illawarra Shoalhaven	Regional and rural	49,791 (15.73)	121 (31.8)
Murrumbidgee	Regional and rural	23,133 (7.31)	42 (11.1)
Southern NSW^d^	Regional and rural	24,483 (7.73)	43 (11.3)
Hunter New England	Regional and rural	118,306 (37.37)	83 (21.8)
South Eastern Sydney	Metropolitan	100,826 (31.85)	91 (23.9)

^a^LHD: local health district.

^b^Number of children aged 0 to 9 years in each LHD (statistics on children aged 2 to 6 years unavailable; Center for Epidemiology and Evidence. HealthStats NSW: Population by Local Health District. 2019).

^c^Remaining participants (n=78) were recruited from areas of NSW that were not specifically targeted for recruitment.

^d^NSW: New South Wales.

### Intervention Preference

#### Ex Ante

At baseline, all participants were asked whether they had a strong preference for how they received health information. Of the 458 participants, 393 (85.8%) stated that they had strong preferences. When asked which delivery medium they preferred, 59.3% (233/393) stated *online*, 28.5% (112/393) stated *written materials*, 11.9% (47/393) stated *telephone calls*, and 0.3% (1/393) stated that they did not know.

#### Ex Post

When asked in the postintervention process evaluation if they would have preferred to receive the intervention in another way, 30.5% (44/144) of the respondents stated that they would have. This included 44% (35/80) of the randomized arm and 14% (9/64) of the preference arm. Further details on the ex post intervention preferences of participants by intervention group and study arm are shown in Table 3. The most commonly stated alternative delivery media preferences were digital delivery mediums such as smartphone apps (10/144, 6.9%) or the web (9/144, 6.3%).

**Table 3:** Ex post intervention preferences of participants by intervention and study arm (N=144).

Intervention	Total participants preferring alternative delivery method			Randomized arm						Preference arm			
	Total sample	Participants, n (%)	Participants preferring alternative delivery method				Preferred method stated^a^		Participants preferring alternative delivery method				Preferred method stated^a^
			Total sample		Participants, n (%)			Total sample			Participants, n (%)		
Telephone	48	13 (27)	35		11 (31)	Web-based (n=9) Smartphone app (n=1) Educational materials (n=1)		13			2 (15)	Phone and web-based (n=2)	
Web-based	57	9 (16)	21		6 (29)	Smartphone app (n=2) Podcast (n=1) Telephone (n=1)		36			3 (8)	Smartphone app (n=3) Educational materials (n=1)	
Active control (written materials)	39	22 (56)	24		18 (75)	Telephone (n=3) Face-to-face (n=1) Smartphone app (n=2)		15			4 (27)	Face-to-face (n=2) Smartphone app (n=2) Skype (n=1)	
Total	144	44 (31)	80		35 (44)	N/A^b^		64			9 (14)	N/A	

^a^Not all participants who preferred an alternative delivery method stated what their preference was, and some participants provided >1 option; hence, some numbers do not add up to the total.

^b^N/A: not applicable.

### Intervention Acceptability

The 3-month postintervention process evaluation found that there was a high level of acceptability for all the interventions, with the median overall score for participants being 22.0 (IQR 5.0) out of a possible high score of 25 (Table 4). The highest median overall score was obtained for the telephone intervention (23.0, IQR 4.0), with the web-based (22.0, IQR 5.0) and active control (written materials; 22.0, IQR 4.0) interventions being similar. There was a significantly higher score for the telephone intervention than that of the active control (written materials) regarding overall acceptability (H_1_=8.258; *P*=.004), the intervention being regarded as interesting (H_1_=9.176; *P*=.002), worthwhile (H_1_=8.878; *P*=.003), and having information that participants could act on (H_1_=10.044; *P*=.002). There was also a significantly higher score for the web-based intervention compared with the active control (written materials) about being regarded as worthwhile (H_1_=6.299; *P*=.01) and having information that participants could act on (H_1_=5.548; *P*=.02).

Participants who completed the in-depth telephone interviews commented that the modules or calls were easy to follow. When asked to rate the interventions on a scale of 1 to 10 (1=very easy to 10=extremely hard), they rated the telephone (mean 3.5, SD 2.2) and web-based (mean 3.1, SD 2.4) interventions similarly. Challenges experienced by participants were usually not related to the interventions per se but rather to the implementation of changes with their children.

**Table 4:** Participant feedback on intervention acceptability from the 3-month postintervention questionnaire (N=144).

	Web-based, median (IQR)				Telephone, median (IQR)				Active control (written materials), median (IQR)				All participants, median (IQR)		
	Randomized (n=21)	Preference (n=15)	All web-based (n=38)	Randomized (n=35)		Preference (n=13)	All telephone (n=48)	Randomized (n=15)		Preference n=23)	All written (n=38)	Randomized (n=79)		Preference (n=64)	Total participants (n=143)
Program was interesting^a^	4.0 (1.0)	5.0 (1.0)	5.0 (1.0)	5.0 (1.0)		5.0 (1.0)	5.0^b^ (1.0)	4.0 (0.0)		4.0 (1.0)	4.0 (1.0)	4.0 (1.0)		5.0 (1.0)	4.0 (1.0)
Program was easy to understand^a^	5.0 (1.0)	5.0 (1.0)	5.0 (1.0)	5.0 (1.0)		5.0 (0.0)	5.0 (1.0)	5.0 (1.0)		5.0 (1.0)	5.0 (1.0)	5.0 (1.0)		5.0 (1.0)	5.0 (2.0)
Program was relevant to family^a^	5.0 (1.0)	4.0 (1.0)	4.0 (1.0)	4.0 (1.0)		4.0 (1.0)	4.0 (1.0)	4.0 (1.0)		5.0 (1.0)	4.0 (1.0)	4.0 (1.0)		4.0 (1.0)	4.0 (1.0)
Program was worthwhile^a^	5.0 (1.0)	5.0 (1.0)	5.0^b^ (1.0)	5.0 (1.0)		5.0 (1.0)	5.0^b^ (1.0)	4.0 (1.0)		4.0 (2.0)	4.0 (1.0)	4.0 (1.0)		5.0 (1.0)	4.0 (1.0)
Could act on information^a^	5.0 (1.0)	4.0 (1.0)	4.5^c^ (1.0)	4.0 (1.0)		5.0 (1.0)	4.5^b^ (1.0)	4.0 (0.0)		4.0 (1.0)	4.0 (0.0)	4.0 (1.0)		4.0 (1.0)	4.0 (1.0)
Overall score^c^	24.0 (5.0)	22.0 (5.0)	22.0 (5.0)	23.0 (4.0)		23.0 (4.0)	23.0^b^ (4.0)	20.0 (3.0)		22.0 (6.0)	22.0 (4.0)	22.0 (5.0)		22.0 (5.0)	22.0 (5.0)

^a^Likert scale of 1 to 5 for each question.

^b^Significant difference between intervention and control (*P*<.05).

^c^Overall score was the sum of all scores (possible range 5-25).

### Web-Based Intervention

#### Overview

Almost all web-based participants in the postintervention process evaluation suggested that the number of modules (ie, n=6) was appropriate (53/56, 95%) and that the length of each module was appropriate (55/57, 96%):

Yeah, I think it was just right. There was a couple of busy weeks for myself so I may have done part of the module each day over the week and then I still had plenty of time to implement my goals.

Participant 2, mother of boy aged 4 years

The web-based intervention comprised the following components: written information, interactive activities, goal setting, videos and quizzes, and an optional Facebook group. Participants in the postintervention process evaluation suggested that the most useful intervention components were written information (23/57, 40%), interactive activities (eg, planner, recipe modification, and label reading; 15/57, 26%), and goal-setting components (13/57, 23%). In contrast, the 10 qualitative interviews for the web-based intervention suggested that goal setting, videos, and quizzes were the most useful components:

My favourite part was the goal setting. It made you reflect, or it was new information to you and actually saying what you’re going to do. It did create a goal for the next couple of weeks and held me accountable to that.

Participant 2, mother of boy aged 4 years

I guess the content. It kind of gave you a plan. The quizzes I suppose I could redo them so I could refresh and put them in place. I could go back and try this. It was kind of like a tool you could go back to.

Participant 20, mother of boy aged 5 years

Of the web-based intervention content (which focused on healthy snacks, healthy meals, physical activity, screen time, and sleep), the highest proportion of participants perceived healthy eating or healthy snacks content as the most useful (27/57, 47%, and 13/57, 23%, respectively) in the postintervention process evaluation. This was supported by the findings from the qualitative part of the study, with most participants stating that healthy eating and physical activity modules were the most useful. The least useful modules were considered to be sleep and screen time as participants thought they had already established good practices in these areas:

Before the program I was really struggling to come up with ideas of healthy snacks. Doing it gave me some more ideas, taught me how to read labels and work out what was healthy and what wasn’t healthy. It also reinforced that most of what I was doing was right but giving me a few extra tricks, I guess.

Participant 11, mother of girl aged 4 years

I think the physical activity...Because they had some ideas, things I could play with the children in the yard. I really like this they do play in the yard but just in case they get bored I have some things I can do with them. I found that helpful.

Participant 13, mother of girl aged 4 years

#### Telephone Intervention

Most participants in the postintervention process evaluation suggested that the number of calls in the telephone intervention was appropriate (40/48, 83%) and that the length of the calls was appropriate (44/48, 92%):

Yeah, I mean they didn’t drag on. They didn’t make any points unnecessarily or whatever.

Participant 17, mother of boy aged 2.5 years

Participants in the postintervention process evaluation reported that the most useful intervention components of the telephone intervention were the verbal information (20/48, 42%), guidebook (15/48, 31%), and goal setting (10/48, 21%). Regarding the useful content, most participants (38/48, 79%) reported that healthy eating was the most useful. These data are supported by the data from the qualitative part of the study with participants, suggesting that the guidebook and goal setting were the most useful intervention components, and the healthy eating content was the most useful. The least useful content was related to screen time and sleep:

I love the guidebook. I thought it was great. Just having that reference, I would look through it before our phone call. I could follow on when having the phone call.

I think the accountability side of it. You would pick the goals and then have someone call and follow up and say how are you going with that. That made you think if you hadn’t been focussing on it you thought yeah I should be doing more in terms of working towards that goal.

Participant 17, mother of boy aged 2.5 years

Participants stated that they benefited from knowledge regarding the amount of physical activity required, goal setting, implementing changes as a family, encouragement of family meals, healthy eating tips, practical advice, and support for implementing changes:

The other really helpful thing was thinking about how to encourage good eating behaviours like mealtime behaviours, sitting with the family. That’s something we changed as well. We used to make my son eat separately. Now from time to time when we can we sit and eat as a family to model the good eating behaviours.

Participant 17, mother of boy aged 2.5 years

In terms of that yeah. Getting her out on a bike getting her out walking. Really promoting more active play outside because it’s not what she would normally tend to show interest towards. That I think has been a lifestyle change for us; it’s something we’ve implemented and stuck to.

Participant 7, mother of girl aged 4 years

#### Active Control (Written Materials)

Approximately three-fourth of the participants in the postevaluation process evaluation suggested that the active control (written materials) components were appropriate in terms of the number of resources and amount of information included (20/27, 74%, and 19/27, 70%, respectively). This was supported by qualitative interview data:

Yeah, I think it was great. Well I think with the exercise bit it was quite good to see what’s considered exercise as well. I made changes. I’ve used the tips for the lunchboxes I guess so I yeah I did use some of these ideas.

Participant 3, mother of girl aged 5 years

I feel just increasing vegetable and fruit intake and making meals a bit more fun. I think that’s probably the main thing that we’ve taken from it. Also, enjoying outdoor activities.

Participant 14, mother of boy aged 3 years

### Fidelity

Significantly more participants in the telephone arm completed the intervention than those in the web-based arm (47/95, 50%, vs 57/218, 26.1%, respectively; *P*<.001). When considering participants who completed the intervention within a 20-week timeframe, there was no significant difference between the 2 groups, with 33% (31/95) of participants completing the telephone intervention and 25.7% (56/218) of participants completing the web-based intervention within 20 weeks. There was no significant difference in intervention completion between the randomized and preference groups. Within the web-based intervention, out of 218 participants, 105 (48.2%) joined the optional Facebook group.

### Withdrawal

Although there was a greater proportion of total withdrawals (including active and passive withdrawal) in the web-based versus telephone group (161/218, 73.9%, vs 47/95, 50%; *P*<.001), there was a significantly higher proportion of participants in the telephone intervention group who actively withdrew from the intervention than those in the web-based intervention group (19/95, 20%, vs 4/218, 1.8%; *P*<.001). There was no significant difference in withdrawal rates between the randomized and preference participants.

## Discussion

### Principal Findings

A comprehensive process evaluation of the *Time for Healthy Habits* translation study of 2 remotely delivered healthy eating and active living interventions and an active control for parents of children aged 2 to 6 years was conducted in this mixed methods study. There was a broad reach of the study across metropolitan, regional, and rural areas of NSW, Australia; however, there were better take-up rates in some areas of the state than in others, with higher participation rates in regional and rural areas than in metropolitan areas. The recruitment effort through the LHDs was substantial. Engagement with existing health promotion staff was crucial for recruitment, as they had established networks with ECEC services to facilitate recruitment. Recruitment was also assisted by having specifically appointed recruitment staff within the target LHDs, who also concentrated largely on ECEC services for recruitment, resulting in 41% (188/458) of the participants being recruited through this channel. This appeared to be particularly useful when the staff attended face-to-face sessions. Social media was another important avenue of recruitment, where 19% (87/458) of the participants found out about the study. After the preference arm was closed, it was perceived that parents were reluctant to be involved as they did not want to be randomized; however, it was difficult to determine whether this was the result of recruitment saturation over time. There was also limited time and capacity for the health promotion staff to be involved in recruitment. Without additional staff resources available in this trial, it is unlikely that recruitment rates would have reached the same level. This is a common issue for translation trials, where dedicated staff are needed to recruit to a program or service, and it is difficult to obtain a sense of true real-world uptake of such interventions. It is possible that future implementation of interventions in LHDs may result in lower uptake rates; however, some parents may also be more inclined to participate in a program if they are not in need to sign up to a research trial.

Although a larger proportion of participants initially preferred a web-based delivery mechanism, the acceptability of both the web-based and telephone interventions was significantly higher than that of the active control (written materials) regarding being worthwhile and containing information that participants could act on. The telephone intervention also demonstrated a significant difference in acceptability compared with the control for the overall acceptability score, taking into account a wider range of acceptability factors. The ease of following the interventions was similar; however, this rating was slightly better for participants who received the web-based intervention than for those who received the telephone intervention. Regarding the components of the intervention, the web-based participants stated that the text information and goal setting were the most useful, whereas the telephone participants felt that the verbal information and guidebook were the most useful. The usefulness of the text and guidebook information may be influenced by the sample being highly educated, and this may not be generalizable to lower socioeconomic populations where literacy levels are known to be lower [21]. Less than half of the participants receiving the web-based intervention joined the optional Facebook group, and similar to previous studies that have used Facebook as a component of an intervention [22], engagement in the discussion was quite low.

Regarding content, the healthy eating aspects were the most useful across all interventions, with sleep and screen time being regarded as the least useful as they felt these were areas in which their children were already doing well, which is fairly consistent with the current evidence concerning these behaviors in that more young children are meeting the movement behavior guidelines than the dietary guidelines, with vegetable intake, in particular, being very low [1]. Past research also indicates that many parents perceive that their young children are naturally active [23]. However, there is still a great need for improvement in relation to physical activity, as the proportion of children meeting the guidelines drops from 75% at the age of 2 to 3 years to 43% at the age of 4 to 8 years [1]. A small number of participants indicated that they regarded limiting screen time as important. The participant interviews were largely conducted before the COVID-19 pandemic restrictions in 2020. Studies investigating the health behavior habits of children during lockdown periods have indicated that screen time has become a considerable concern that could lead to long-term increased use [24]. Therefore, it is possible that parental concern regarding screen time may have increased since this time and could be in greater need of focus in the future.

For participants in both interventions, engagement declined over time, particularly for those receiving web-based intervention. It is important to address reduced engagement and participation levels, as implementation levels have been demonstrated to have an impact on study outcomes [25]. As suggested in previous studies, offering participants flexibility and choice of delivery medium may assist in uptake and engagement in interventions [26], and it may be worthwhile to consider alternate delivery options in future studies. Most participants who identified an alternate delivery means specifically identified a smartphone app; however, it should be noted that previous research has indicated that apps can also have high attrition rates [27]. Some participants commented that a combination of delivery mechanisms such as telephone and web-based or telephone and smartphone apps would be preferable. Many of the participants who completed the interviews stated that they would also like to receive ongoing support to help embed knowledge and sustain their practices. It is likely that different mediums are a matter of individual preference, and providing multiple options to access interventions may be beneficial when scaling to a population level; however, the practicalities and costs of offering multiple mediums would need to be considered carefully. The only significant outcome of this study was in relation to children’s dietary intake of noncore foods. This may be because the healthy eating modules or calls were completed first and by a higher proportion of participants. By the same token, the reason for no significant outcomes for physical activity, screen time, or sleep may be as these topics were covered later, and as the interventions needed to be completed sequentially and engagement dropped off over time, fewer participants completed these calls or modules. Completing the calls and modules sequentially may not be suitable for all participants, and it may be preferable to allow participants to choose their main topics of interest or concern and complete them first.

There was a high withdrawal rate for the interventions, particularly the web-based intervention. Although significantly more telephone participants actively withdrew from the intervention, this may have been because of the nature of the intervention, whereby regular telephone contact was required, and thus, participants needed to actively withdraw if they did not want to receive further phone calls. Previous translation trials have reported challenges with withdrawal and retention [12,28,29]. Several parents cited a lack of time to participate, a common barrier highlighted in previous studies involving parents [26]. There was difficulty contacting some participants receiving the telephone intervention, and although several attempts were made, some participants could not be contacted.

Parents with a high level of education were overrepresented in the study, a challenge that has been described in other similar studies [30]. A previous study, Healthy Habits, Happy Homes Scotland, effectively engaged lower socioeconomic families, with 65% of the participants living in the most deprived areas. This was achieved using participatory and inclusive strategies, making strong connections with parents and supporting organizations, and coproducing the intervention [11]. It is important that interventions are designed to be acceptable and accessible to lower socioeconomic families so that they are adequately represented in studies, or there is a danger of the gap in health outcomes widening [31]. Other translation studies on older children have effectively reached lower socioeconomic families, with most of the participants recruited through schools or self-referrals [12,13]. However, families from low socioeconomic backgrounds can be less likely to complete these interventions [13]. Given the successful recruitment of participants from ECEC services in this study, focusing on ECEC services in specific postcodes with a high level of social disadvantage may be an effective strategy for engaging families from lower socioeconomic backgrounds in the future. There was also a much higher representation of mothers than fathers. Often, mothers are the primary caregivers of children at this age, which is unsurprising. Previous research has also found that one of the barriers to fathers participating in research is the relative lack of time and availability relative to mothers [32]. In addition, an inclusion criterion was that the child needed to live with the parent for at least 4 days per week to have the opportunity to influence child behaviors, which may have prevented the participation of some fathers with joint custody arrangements. There is evidence that fathers can have a profound influence on the dietary intake and physical activity habits of their children; therefore, it is important that future studies consider the engagement of fathers and ensure that interventions are relevant and accessible to them [33,34].

### Strengths and Limitations

A strength of this study was the use of a comprehensive combination of quantitative and qualitative methods to evaluate the process of delivering the interventions, which attempted to obtain diverse representations across the LHDs involved. The qualitative interviews were conducted by a separate research organization; therefore, participants may have been more likely to provide more honest responses to the questions asked. The evaluation was limited by the modest proportion of participants (144/458, 31.4%) who completed the process evaluation questionnaire after the intervention. The sample may have been biased toward those who completed the intervention, and the views of participants who did not complete the interventions may differ from those who completed the interventions. Although all participants were asked to complete the questionnaire around 3 months after baseline, regardless of whether they had completed the interventions, most participants who completed the questionnaire had finished the interventions. Similarly, despite efforts to engage participants who had not completed the interventions in the qualitative interviews, all participants who completed the interviews had completed the interventions; therefore, it was difficult to ascertain the specific reasons for the noncompletion of the interventions. Although the interviews were conducted by a separate research organization, it is possible that participants may not have given their honest opinions. Parents with a university education were overrepresented in the study; therefore, these process evaluation results may not be representative of the general population. Finally, this study was conducted during a period that encompassed the height of the 2020 COVID-19 pandemic restrictions in NSW from mid-March to late May 2020, when most school children were at home learning remotely, ECEC services were encouraging children to stay at home, and many parents were working from home. Recruitment of participants was likely affected by these restrictions, and anecdotal reports indicate that some parents found the additional time pressures during this period difficult, and completion of the interventions and the implementation of behavior changes may have been affected as a result.

### Conclusions

This mixed methods process evaluation demonstrated a high level of acceptability of all interventions but a strong participant preference for the web-based intervention. Although the web-based intervention was the most preferred, fidelity was lower, and dropout was higher (although more participants actively dropped out of the phone intervention). Despite the high rate of acceptability of the interventions, refinement of the delivery model appeared preferable to some participants. However, any potential modifications to existing interventions should ensure that outcomes are not compromised. The results of this study highlight the strengths and weaknesses of these remotely delivered interventions and offer important aspects for policy makers and practitioners to consider along with the main study outcomes.

## Appendix

Multimedia Appendix 1 Local health district staff recruitment survey.

Multimedia Appendix 2 Process evaluation questions.

Multimedia Appendix 3 Qualitative telephone interview questions.

## Abbreviations

ECEC: Early Childhood Education and Care
LHD: local health district
NSW: New South Wales
RCT: randomized controlled trial
