# Supplementary material for: Telephone and Web-Based Delivery of Healthy Eating and Active Living Interventions for Parents of Children Aged 2 to 6 Years: Mixed Methods Process Evaluation of the Time for Healthy Habits Translation Trial
Source: J Med Internet Res. 2022 May 26;24(5):e35771. doi: 10.2196/35771 (PMC9185338; doi:10.2196/35771)
Supplement: Multimedia Appendix 1 [file jmir_v24i5e35771_app1.docx]

**Supplementary File 1: Local Health District (LHD) Staff Recruitment Survey**

1. What were the channels of recruitment that were most effective within your LHD? (please specify if these channels were face-to-face, or via email, phone etc)
2. What contributed to the success of those recruitment channels?
3. What were the channels of recruitment that were least effective in your LHD? (please specify if these channels were face-to-face, or via email, phone etc)
4. What contributed to the lack of effectiveness of these channels?
5. What would you do differently if you were to use these methods again?
6. What were the main challenges that you faced with recruitment?
7. How could you have overcome these challenges?
8. What other forms of support would have been helpful to your recruitment efforts?
9. Other than the dedicated staff member who actively worked on recruiting participants in your LHD, were there other Health Promotion Officers, or other key stakeholders who assisted with recruitment in your area? Who were they, and how did they assist?
10. If you have any other feedback, please enter below
