# Supplementary material for: Telephone and Web-Based Delivery of Healthy Eating and Active Living Interventions for Parents of Children Aged 2 to 6 Years: Mixed Methods Process Evaluation of the Time for Healthy Habits Translation Trial
Source: J Med Internet Res. 2022 May 26;24(5):e35771. doi: 10.2196/35771 (PMC9185338; doi:10.2196/35771)
Supplement: Multimedia Appendix 2 [file jmir_v24i5e35771_app2.docx]

**Supplementary File 2: Process Evaluation Questions**

| **Telephone** | **Online** | **Written (control)** |
| --- | --- | --- |
| T1. Do you think that six phone calls is an appropriate number of calls (Response Options: Yes, No, Don’t know) Show Q T2 if No | O1. Do you think that six modules is an appropriate number of modules? (Response Options: Yes, No, Don’t know) Show Q O2 if No | W1. Do you think the 10 factsheets and 1 summary booklet is an appropriate number of resources? (Response Options: a) Yes b) No I would have preferred more resources c) No I would have preferred less resources d) Don’t know) |
| T2. Would you have preferred more calls or fewer calls? (Response Options: More, Fewer, Don’t know) | O2. Would you have preferred more or fewer modules? (Response Options: More, Fewer, Don’t know) | W2. Do you think the resources had an appropriate amount of information? (Response Options: a) Yes b) No I would have preferred more information c) No I would have preferred less information d) Don’t know) |
| T3. Do you think that phone calls were of an appropriate length of time for you? (Response Options: Yes, No, Don’t know) Show Q T4 if No | O3. Do you think the modules were an appropriate length? (Response Options: Yes, No, Don’t know) Show Q O4 if No |  |
| T4. Would you have preferred longer or shorter calls? (Response Options: Longer, Shorter, Don’t know) | O4. Would you have preferred longer or shorter modules? (Response Options: Longer, Shorter, Don’t know) |  |
| T5. The program aspect that was most useful to you was…(Response Options a) The Guidebook b) Verbal information c) Goal setting d) Activities e) Meal planners f) Don’t know) | O5. Do you think the time allocated to complete the modules was appropriate? (Response Options: Yes, No, Don’t know) Show Q O6 if No |  |
| T6. The module that was most useful to you was….(Response Options a) Healthy eating b) Physical activity c) Screen time d) Sleep e) Don’t know) | 06. Would you have preferred more time or less time to complete the modules? (Response Options: More time, Less time, Don’t know) |  |
|  | O7. The program aspect that was most useful to you was….(Response Options a) Text information b) Videos c) Goal setting d) Activities (e.g. planner, recipe modification, label reading, e) Facebook group f) Quizzes g) Don’t know) |  |
|  | O8. The module that was most useful to you was…Response Options a) Healthy meals b) Healthy snacks c) Physical activity d) Screen time e) Sleep f) Don’t know) |  |
| A1. The program was interesting (Response Options: 1=Strongly disagree, 2=Disagree, 3=Neutral, 4=Agree, 5=Strongly agree) | | |
| A2. The program was easy to understand (Response Options: 1=Strongly disagree, 2=Disagree, 3=Neutral, 4=Agree, 5=Strongly agree) | | |
| A3. The program content was relevant to your family (Response Options: 1=Strongly disagree, 2=Disagree, 3=Neutral, 4=Agree, 5=Strongly agree) | | |
| A4. Participation in the program was worthwhile (Response Options: 1=Strongly disagree, 2=Disagree, 3=Neutral, 4=Agree, 5=Strongly agree) | | |
| A5. The information I received was something I could act on (Response Options: 1=Strongly disagree, 2=Disagree, 3=Neutral, 4=Agree, 5=Strongly agree) | | |
| A6. Have you talked about this program with any extended family members (e.g. grandparents) or carers? (Response Options: Yes, No, Don’t know) | | |
| A7. Have you talked about this program with any other parents who have a child aged 2- to 6-years? (Response Options: Yes, No, Don’t know) | | |
| A8. Having completed the program, would you have preferred for the information to be delivered in another way? (Response Options: Yes, No, Don’t know) | | |
| A9. In which format would you rather receive the advice? (Response Options: a)Online program b) telephone counselling c) Educational materials d) Smartphone app e) Face-to-face f) Skype g) Other h) Don’t know) | | |
| A10. Why would you prefer this method of delivery? | | |
| A11. Do you have any other feedback about the program? | | |
