# Supplementary material for: Telephone and Web-Based Delivery of Healthy Eating and Active Living Interventions for Parents of Children Aged 2 to 6 Years: Mixed Methods Process Evaluation of the Time for Healthy Habits Translation Trial
Source: J Med Internet Res. 2022 May 26;24(5):e35771. doi: 10.2196/35771 (PMC9185338; doi:10.2196/35771)
Supplement: Multimedia Appendix 3 [file jmir_v24i5e35771_app3.docx]

**Supplementary File 3: Qualitative Telephone Interview Questions**

| **Overall experience**  1. Can you please tell me why you were initially interested in the program?  2. What were your initial expectations about the program?  [Prompt: What were you hoping to get out of the program?]  3. Were these expectations met? In what way were they met or not met? | |
| --- | --- |
| **Telephone intervention-specific experiences**  As you would recall the phone call program involved participating in 6 phone calls. The first four phone calls focused on healthy eating, the fifth phone call focused on physical activity and screen time, and the sixth phone call focused on sleep.  4. Based on your experience with the program, what would you suggest was the most useful phone call?  *[Prompt and might need to provide some additional information to refresh memories: healthy eating, physical activity, screen time and sleep]*  5. Can you please tell us why this phone call was the most useful?  6. What information did you find helpful in this phone call compared to the others?  7. Was the content in this phone call more practical than that in the other phone calls?  8. Which phone call did you find the least useful or helpful?  9. Can you please tell us why you found this phone call the least useful or helpful?  *[Prompt – most relevant, new information, most applicable, was at the beginning]*  In addition to the phone calls you received a guidebook and some meal planners. You may also recall that throughout the program you were asked to participate in a number of activities and set a number of goals related to the content.  10. Based on your experience with the program, was the aspect of the program that was useful to you.  *[Prompt –phone calls, guidebook, meal planners, activities, and goal setting]*  11. Can you please explain why this aspect of the program was the most useful to you (as opposed to [mention the other components of the program])?  12. What aspect of the program was least useful/helpful to you?  13. Why did you consider this aspect of the program to be the least useful?  The program was on average 16 weeks in length. Some participants took slightly shorter or longer to complete the program  14. Would you suggest that this was an appropriate length of time for a program?  15. Why or why not? [Prompt: would you have preferred a different schedule for the 6 calls (Currently these is approx. 1 call per fortnight)?]  16. Did your interest in the content of the program change over time?  17. Can you suggest ways to keep parents interested in the program from the start to finish?  *[Prompt: times of low and high interest, loss of motivation over time or during certain modules]*  Based on your experience with the program, was the length of the phone calls appropriate, or too short or too long?  18. Can you please tell us a little more about why you thought the phone calls were [too long or too short]? [Prompt if calls are too long: would you prefer to have shorter calls, more frequent calls?]  On a scale of 1 to 10 how easy or hard was it for you to complete the program (with 1 being very easy and 10 being extremely hard)?  19. Were there aspects of the program that you found difficult or frustrating?  20. Why were these aspects of the program particularly hard? (if not already answered in above question) Any suggestions on how this could be improved?  21. Did you find the health coach to be helpful and supportive?  22. Did you feel comfortable talking to your coach? Why or why not?  23. In general, is there anything about the program that you feel could be improved or changes?  24. What might these be and why might how would you like them changed? *[Prompt– length/duration, frequency of contact, content, follow-up]* | **Online intervention-specific experiences**  As you would recall the online program involved participating in 6 online modules. The first module introduced the program, the second and third module focused on healthy meals and healthy snacks. The fourth module looked at physical activity and the fifth module focused on screen time. The last module spoke about sleep.  4. Based on your experience with the program what was the most useful module?  5. Can you please tell us why this module was the most useful?  6. What information did you find helpful in this module compared to the others?  7. Was the content in this module more practical than that in the other modules?  8. Which module did you find the least useful or helpful?  9. Can you please tell us why you found this module the least useful or helpful?  *[Prompt – most relevant, new information, most applicable, was at the beginning]*  You may also recall that throughout the program there were videos to watch, online activities and quizzes to complete and a goal setting section at the end of each module. You might also remember taking part in a closed Facebook group.  10. Based on your experience with the program what was the aspect of the program that was useful to you?  11. Can you please explain why this aspect of the program was the most useful to you (as opposed to [mention the other components of the program – text information, videos, activities, goal setting, feedback, Facebook group])?  12. What aspect of the program was least useful/helpful to you?  13. Why did you consider this aspect of the program to be the least useful?  The program was on average 12 weeks in length. Some participants took slightly shorter or longer to complete the program  14. Would you suggest that this was an appropriate length of time for a program?  15. Why or why not?  16. Did your interest in the content of the program change over time?  17. Can you suggest ways to keep parents interested in the program from the start to finish?  *[Prompt: times of low and high interest, loss of motivation over time or during certain modules]*  18. Based on your experience in the program would you suggest that the time to complete each module was just right, too long or too short?  19. Can you please tell us a little more about why you thought the time to complete each module was [too long or too short]?  On a scale of 1 to 10 how easy or hard was it for you to complete the program (with 1 being very easy and 10 being extremely hard)?  20. Were there aspects of the program that you found difficult or frustrating?  21. Why were these aspects of the program particularly hard? (if not already answered in above question) Any suggestions on how this could be improved?  Thanks for answer all of these questions. We are nearly at the end.  22. In general, is there anything about the program that you feel could be improved or changes?  23. What might these be and why might how would you like them changed?  *[Prompt– length/duration, frequency of modules, content, follow-up)* |
| **Intervention impacts**  The last section of the interview focuses on changes that you and your family may or may not have made as a result of participating in the program.  25. Overall, do you feel that you and/or your family have benefited from the program? How?  26. Have you been able to make any lifestyle changes as a result of participating in the program? *[Prompt: Changes diet, physical activity, sedentary behaviours, sleep]*  [If YES]  27. Can you please tell me about these changes? [Probe: What changes have you and/or your family made?]  28. How did the program help you make these changes?  *[Prompt: Do you think you would have made these changes without the program?]*  29. Did you experience any difficulties or challenges when trying to make these changes? [If yes] Can you please tell me about them? How did you overcome them?  30. Are you planning to maintain the changes that you have made now that the program has finished?  [If YES]  31. How are you planning to maintain these changes? What do you think will help you do this?  32. Do you think your family will continue to make changes?  33. What difficulties or challenges, if any, do you think you will experience? What support would you ideally like to receive?  34. [If NO] Why not?  35. Would you recommend the program to other families? Why or why not?  36. Have you recommended the program or shared the materials with people beyond outside of your family in the community (for example, wider family or friends in your community)?  [If yes]  37. How many people outside your immediate family did you talk to? Who were they?  38. What did you share with them? How did these people respond?  *[prompt: they signed up to the program, grandparents used some of the information, friends viewed the information]* | |
